# ‘Social screens’ and ‘the mainstream’: longitudinal competitors of non-organized physical activity in the transition from childhood to adolescence

**DOI:** 10.1186/s12966-019-0908-0

**Published:** 2020-01-14

**Authors:** Byron J. Kemp, Anne-Maree Parrish, Dylan P. Cliff

**Affiliations:** 1 Early Start, University of Wollongong, Northfields Avenue, Wollongong, NSW 2522 Australia; 2 School of Health and Society, Faculty of Social Sciences, University of Wollongong, Northfields Avenue, Wollongong, NSW 2522 Australia; 3 Illawarra Health and Medical Research Institute, University of Wollongong, Northfields Avenue, Wollongong, NSW 2522 Australia; 4 School of Education, Faculty of Social Sciences, University of Wollongong, Northfields Avenue, Wollongong, NSW 2522 Australia

**Keywords:** Adolescent, Leisure time physical activity, Active play, Health promotion, Social marketing, Segmentation

## Abstract

**Background:**

Physical activity (PA) tends to decline during late childhood and adolescence. In Australia, this decline has been shown to occur particularly in non-organized PA (e.g. active play and informal sport). Using a social marketing approach, segments of youth may be identified and targeted based on their profile of alternative activities that compete with non-organized PA during the transition to adolescence. The objectives of this study were to identify and describe segments of youth whose participation in non-organized PA declined between 11 and 13 years, based on changes in other potential competing activities during this period.

**Methods:**

Data were sourced from Waves 4 and 5 of the Longitudinal Study of Australian Children. Participation in non-organized PA and thirteen alternative activities (e.g. video games, homework, sleep) were measured using 24-h time-use diaries. Analyses were limited to participants whose non-organized PA had declined between 11 and 13 years (*n* = 1043). Two-stage cluster analysis was conducted and segments were described using chi-square and t-tests.

**Results:**

Among the analytic sample, average non-organized PA participation declined by 87 min/day between 11y and 13y (*p* < 0.001). Two segments were identified (κ = 0.66). The ‘Social Screens’ segment (*n* = 143) had large increases in texting, emailing and social media use (+ 56 min/day, *p* < 0.001) and other internet use (+ 32 min/day, *p* < 0.001). Conversely, ‘the Mainstream’ segment (*n* = 900) had smaller increases in a wider range of activities, including other PA (organized PA, active transport, active chores/work) (+ 16.0 min/day, *p* < 0.001), homework/study (+ 9.5 min/day, *p* < 0.001) and electronic gaming (+ 6.7 min/day, *p* < 0.05). ‘Social Screens’ were more likely to attend public school, live in urban areas and have more advanced pubertal development (girls only). ‘The Mainstream’ were more likely to participate in PA and out-of-school activities.

**Conclusions:**

The ‘Social Screens’ segment had a much larger increase in texting, emailing, social media and other internet use, and lower participation in overall PA and out-of-school activities, compared with ‘the Mainstream’. Future research may trial PA promotion strategies to replace benefits that this segment may seek in competing activities (e.g. social PA apps).

## Background

Participation in physical activity (PA) is favourably associated with a range of health outcomes for children and youth [1]. However, combined data from 105 countries suggest that the majority of youth are not meeting global PA recommendations [2, 3]. PA participation is prone to decline during late childhood and adolescence [4], and recent studies have explored whether this decline occurs in specific domains of PA [5]. In particular, a recent Australian longitudinal study reported a sharp decline in the domain of non-organized PA between 11 and 13 years [6]. Non-organized PA includes activities that tend to be unstructured, freely-chosen, spontaneous and occurring for their own sake (e.g. active play and informal sport) [7]. Non-organized PA accounted for the bulk of the decline in overall PA participation among this cohort between 11 and 13 years; while other domains of PA remained stable (organized PA) or increased slightly (active transport and active work/chores) [6]. This indicates that declines in non-organized PA among this cohort were not necessarily offset by sufficient increases in other domains of PA. Therefore, participation in non-organized PA may be explored as a potential behavioral target for intervention in the Australian context.

One approach to PA promotion is the use of social marketing [8], a practice which seeks to “develop and integrate marketing concepts with other approaches to influence behaviours that benefit individuals and communities for the greater social good” [9]. One aspect of the social marketing framework is competition analysis, which seeks to determine factors that compete with the target behavior for the time and attention of the audience [10]. This information may then be used to promote the benefits of the target behavior and minimize the costs, relative to competing behaviors [10]. This approach may also enhance the process of market segmentation, by highlighting differences in competing behaviors across segments [11].

Despite these potential benefits, the longitudinal competitors of non-organized PA are relatively unknown. A systematic review [12] and subsequent literature search revealed that only three studies of adolescent time-use clusters have included the domain of non-organized PA [13–15]. Clusters generally contrasted non-organized PA with screen time, although the extent of displacement between these activities over time was unclear due to the cross-sectional design of studies [13–15]. Therefore, the objectives of the present study were to identify and describe segments of youth whose participation in non-organized PA declined between 11 and 13 years, based on changes in other potential competing activities during this period.

## Methods

### Participants and procedures

The present study included data from the Kindergarten cohort Longitudinal Study of Australian Children (LSAC), an ongoing cohort study managed by the Australian Department of Social Services [16]. The study began in 2004 with a nationally-representative sample of 4983 children aged 4–5 years [16]. A two-stage clustered sampling strategy was used to recruit participants within postcodes from the national Medicare database [16]. Children were eligible for the study if they were born between March 1999 and February 2000 [16]. Participants have been followed-up every two years since baseline via mail, phone and interviewer visits. The present study draws on data from Wave 4 (2010) and Wave 5 (2012) of the study, when the average age of children was 11 years and 13 years respectively. Data collection procedures for LSAC were approved by the Australian Institute of Family Studies Ethics Committee and informed consent was provided by participants [17]. The present study was approved by the University of Wollongong Human Research Ethics Committee (2017/275).

## Measures

### Time-use variables

Time-use diaries (TUDs) were used in both waves to measure the duration of non-organized PA and 13 potential competing activities over a 24-h period. Self-report instruments such as TUDs are often used in studies that focus on PA domains because the context of PA cannot easily be determined via more objective methods such as accelerometry [18]. The use of TUDs also ensured that all clustering variables were measured in the same units (minutes). At both time-points, LSAC participants were mailed a paper diary with instructions to record their activities for a 24-h period on the day before their interview [19]. The diary had an open-ended format that allowed participants to record activities in their own words. Participants who attended school on the day before their interview were instructed to record activities that occurred during recess and lunch but not during school lessons (including PE lessons). Later during the home interview, the TUD data for each participant was entered by interviewers using a predetermined coding framework [19]. Interviewers were also trained to identify gaps in the diaries and prompt participants for additional information as needed [19].

Definitions of non-organized PA and the 13 other activities used as potential clustering variables in this study are provided in Table 1. In the present study, the duration of these activities was extracted from LSAC datasets by the lead author (BK). Activities were included in the study if they were measured using similar TUD categories and if at least 5% of the sample had participated in the activities in either wave.

**Table 1:** Description of non-organized PA and the variables used in initial cluster analysis

Variable	Description
Non-organized physical activity (outcome)^a^	Ball games, riding bike/scooter/skateboard for leisure, skipping, running, etc. (the overall term ‘unstructured active play’ was adopted in Wave 5).
Other physical activity^a^	Organized PA (organized team sports, organized individual sports)
	Active transport (travel by foot, bike, scooter, skateboard, etc.)
	Active chores/work (e.g. gardening, walking pets, making beds)
Daily living activities	Personal care (bathing, cleaning teeth, getting ready, etc.)
	Health and medical care (doctor, dentist, allied health, etc.)
	Non-active chores (cooking, washing dishes, caring for siblings, etc.)
	Non-active travel (car, bus, train, etc.)
Sleeping/napping	Sleeping^b^, napping
Homework/study	Homework, tutoring, private lessons (e.g. music lessons)
School lessons	School lessons
Shopping	Shopping (excluding internet shopping)
Music for leisure	Playing/listening to music for leisure
Reading for leisure	Reading/being read to for leisure
Electronic gaming	Playing electronic games on a computer or console
Television (TV)/movies	Watching TV, DVDs or going to the cinema
Verbal communication	Talking face-to-face, on the phone or via Skype/webcam^c^
Texting/emailing/social media	Texting, emailing, instant messaging, spending time on social networking sites^d^
Other internet use	General internet browsing, downloading/uploading content, internet shopping, etc.

a.More information about the distinction between domains of PA has been provided in Additional File 2.

b.The duration of sleeping was imputed as the difference between sleep time and wake time within the 24-h period.

c.Skype/webcam use was included in verbal communication, as opposed to internet use because it is a form of synchronous communication and is associated with more affiliative benefits than talking on the phone [20].

d.Texting, emailing and social media use was differentiated from other internet use because it is more socially oriented.

### Other measures

Twenty variables were used to explore the characteristics of the resultant segments at 13 years of age. Additional File 1 provides a description of each variable, including the number of items, response categories, source, and validity and reliability information where available. Variables were grouped according to common marketing segmentation bases [21], including demographic/physical characteristics (e.g. sex, physical health, Indigenous status), geographic characteristics (urban/rural status), psychographic characteristics (e.g. temperament, bullying victimisation) and behavioral characteristics (e.g. participation in out-of-school activities). Measures were generally self-reported by the responding parent during the home interview, although some measures were directly recorded by interviewers (e.g. body mass index).

### Potential confounders

Two potential confounding variables were included in the present study. Firstly, the season of measurement was considered to be a potential confounder because PA and sedentary behavior are prone to seasonal variation [22]. The season of measurement was calculated from the interview date at each wave. Another potential confounder was the ‘type of day’ that had been recorded in the TUD (school attendance/no school attendance). School attendance was included as a variable in the LSAC datasets, and missing data for this variable were imputed based on data reported about ‘school lessons’ in the TUD (it was assumed that participants attended school if ‘school lessons’ were reported in the TUD).

### Analysis

Analyses were conducted using SPSS version 25 (IBM Corporation, Armonk, NY, USA). Effects were considered to be statistically significant at *p* < 0.05. After extracting time-use data, frequency histograms revealed that respondents tended to round their TUD entries to the nearest 5 min. Therefore, the duration of each activity type was consistently rounded to the nearest 5 min for all cases. Change scores were then calculated for the duration of each activity type between 11 and 13 years.

The analyses in the present study were restricted to a subset of cases. Firstly, all cases in the analytic sample had declined in their non-organized PA participation between 11 and 13 years. This allowed cases to be profiled based on the pattern of alternative activities that may have replaced non-organized PA during this period. Secondly, to account for the potential confounding effect of school attendance on the day of the TUD, only those participants with a consistent ‘type of day’ in both waves were included (i.e. two school days or two non-school days).

Cluster analysis was used to identify patterns of change in the time-use variables listed in Table 1. Two-step cluster analysis was used in the present study, as this is commonly used in segmentation studies within the field of social marketing [23]. Input variables were standardized and a log-likelihood distance measure was used. Prior to data analysis, cases were randomly sorted by the last digit of their case identification number to attenuate the potential influence of case order on the final cluster solution, as advised by Norusis [24]. All 13 time-use variables were included in the initial model. Post-hoc tests were used to examine whether the input variables differed significantly across resultant segments [23]. Independent samples t-tests were used for two cluster solutions and one-way analyses of variance (ANOVAs) were used for three-or-more cluster solutions. Input variables that did not differ significantly across segments were removed and the cluster analysis was repeated [23]. This process continued iteratively until segments differed significantly in all input variables. The internal consistency of the cluster solution was then tested by randomly dividing the sample in two and repeating the above process with both halves of the sample [23, 25].

Finally, the characteristics of the final segments were explored using independent-samples t-tests and chi-square tests of independence. The longitudinal changes in each input variable were also explored for each segment using paired-samples t-tests, and effect sizes were used to compare the magnitude of changes between segments.

## Results

**Fig. 1:**
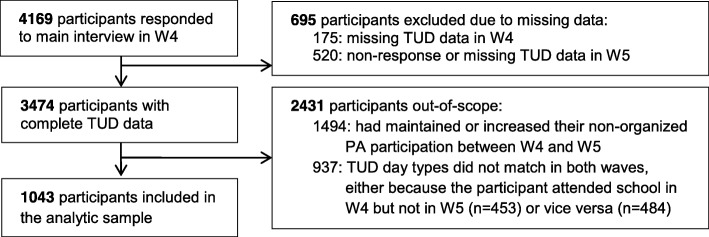
Flowchart showing the reasons for exclusion from the analytic sample

A total of 4169 participants responded to the LSAC main interview in Wave 4. Of these, 1043 participants were included in the analytic sample of the study (25.0%). Some participants were excluded from the study because they had missing data, either at Wave 4 (*n* = 175) or Wave 5 (*n* = 520). Other participants were out-of-scope, either because their participation in non-organized PA had not declined (*n* = 1494) or because they had completed the TUD on different types of days across waves (*n* = 937). Fig. 1 provides a flowchart showing how the analytic sample was determined. The average follow-up duration for the analytic sample was 2.1 years (SD = 0.2 years).

Although the present study included only a subset of cases, the characteristics of the analytic sample were generally similar to the full sample at Wave 4 (see Table 2). The only significant difference was that participants in the analytic sample were more likely to have attended school on the days that the TUDs were recorded (61.5%), compared with the full sample (49.4%) (*p* < 0.001). This is unsurprising because one of the criteria for inclusion in the analytic sample was having consistent TUD day types across waves, and there is a greater probability of two school days being selected than two non-school days. The analytic and full samples did not differ significantly in terms of sex, age, socioeconomic position, Indigenous status, school type, language spoken at home, single parent status, geographical remoteness or season of measurement.

**Table 2:** Characteristics of the analytic and full samples at Wave 4, LSAC data^a^

Variable [wave of measurement]	Analytic sample (*n* = 1043)^b^	Full sample (*n* = 4169)^c^
Males, *n* (%) [w1]	538 (51.6%)	1594 (51.0%)
Age of child, mean (SD) [w4]	10.9 (0.3)	10.9 (0.3)
Family socioeconomic position index, mean (SD) [w4] ^d^	0.0 (1.0)	0.0 (1.0)
Child attends public school, *n* (%) [w4]	642 (62.0%)	2008 (64.6%)
Speaks a language other than English at home, *n* (%) [w4]	84 (8.2%)	251 (8.2%)
Aboriginal or Torres Strait Islander, *n* (%) [w1]	23 (2.2%)	95 (3.0%)
Single-parent family, *n* (%) [w4]	148 (14.2%)	504 (16.1%)
Lives in regional or remote area, *n* (%) [w4]	472 (15.2%)	149 (14.3%)
Attended school on day of TUD, *n* (%) [w4]	641 (61.5%)***	1457 (49.4%)***
Season of measurement, *n* (%) [w4]		
Summer	5 (0.5%)	17 (0.5%)
Autumn	269 (25.8%)	814 (26.0%)
Winter	548 (52.5%)	1564 (50.0%)
Spring	221 (21.2%)	731 (23.4%)

*n* number of participants, *w* wave, *%* proportion of sample, *SD* standard deviation

Bold text indicates statistically significant differences: ***p < 0.001

a.The analytic sample was selected due to having a decline in non-organized PA participation between 11 and 13 years and a consistent ‘type of day’ in the TUD in both waves.

b.Variable-specific missing data for analytic sample: socioeconomic position (*n* = 10), school type (*n* = 8), language spoken at home (*n* = 22), number of parents in home (*n* = 2), remoteness (*n* = 2).

c.Variable-specific missing data for full sample: socioeconomic position (*n* = 25), school type (*n* = 19), language spoken at home (*n* = 83), Aboriginal or Torres Strait Islander status (*n* = 2), number of parents in home (*n* = 3), remoteness (*n* = 18), TUD day type (*n* = 175).

d.The socioeconomic position index was z-scored and ranged from − 5.4 to 2.9 in the overall sample.

Table 3 shows the initial and final cluster solutions of the present study. The final model produced two segments that significantly differed in the uptake in texting, emailing and social media use (*p* < 0.001) and other internet use (*p* < 0.001). No other variables were included in the final model. The model had a silhouette measure of cohesion and separation of 0.8, which is considered to be a good solution [26]. In terms of internal consistency, both split-half models resulted in two segments, defined by differences in the same variables (texting/emailing/social media and other internet use). The vast majority of cases were assigned to the same segment in the original and split-half models (91%), resulting in substantial internal consistency (κ = 0.66, *p* < 0.001) [27].

**Table 3:** Characteristics of initial and final cluster analysis solutions, presented in order from the first model (all variables included) to the final model (only two variables included), LSAC data ^a^

Model characteristics	Model 1	Model 2	Model 3	Final model
Number of clusters/segments	2	3	2	2
Silhouette measure of cohesion and separation	0.1 (poor)	0.0 (poor)	0.5 (good)	0.8 (good)
Predictor importance of variables				
Texting/emailing/social media	1.00	1.00	1.00	1.00
Other internet use	0.50	0.49	0.46	0.39
Other physical activity	0.03	0.03	0.01	–
Shopping	0.15	0.01	–	–
Reading for leisure	0.13	0.01	–	–
TV/movies	0.04	0.01	–	–
Music for leisure	0.04	–	–	–
Electronic gaming	0.04	–	–	–
Homework/study	0.01	–	–	–
School lessons	0.01	–	–	–
Sleeping/napping	0.00	–	–	–
Daily living activities	0.00	–	–	–
Verbal communication	0.00	–	–	–

a.Models were conducted iteratively until segments differed significantly in all input variables. Non-significant variables were excluded from subsequent models.

Table 4 shows the longitudinal changes in the duration of various activities between 11 and 13 years, for the analytic sample and for each segment. Among the analytic sample, the average participation in non-organized PA declined by 87 min/day between 11 and 13 years of age (from 106 min/day to 19 min/day, *p* < 0.001). Segment 1 was labelled ‘Social Screens’ (*n* = 143) due to large increases among this segment in texting, emailing and social media use (+ 56.4 min/day, *p* < 0.001), and a moderate increase in other internet use (+ 32.2 min/day, *p* < 0.001). Segment 2 was labelled ‘the Mainstream’ because this segment included the majority of participants in the analytic sample, and these participants had less extreme increases spread across a wider range of activities, such as other PA (+ 16.0 min/day, p < 0.001), homework/study (+ 9.5 min/day, p < 0.001) and electronic gaming (+ 6.7 min/day, *p* < 0.05). In both segments, significant increases in participation were observed in verbal communication (both p < 0.001), texting, emailing and social media use (both p < 0.001), and daily living activities (‘Social Screens’: p < 0.05; ‘the Mainstream’: *p* < 0.01).

**Table 4:** Longitudinal changes in activity duration between 11y and 13y, overall sample and clusters, LSAC data

Activity	ANALYTIC SAMPLE (*n* = 1043)		SEGMENT 1 (*n* = 143) (‘Social Screens’)		SEGMENT 2 (*n* = 900) (‘The Mainstream’)	
	Mean change (95% CI) (min/day)	Effect size (d)	Mean change (95% CI) (min/day)	Effect size (d)	Mean change (95% CI) (min/day)	Effect size (d)
Non-organized PA	−87.0 (−91.4, −82.5)***	−1.19	−88.4 (−101.1, −75.8)***	−1.16	−86.7 (−91.5, −82.0)***	− 1.20
Texting/emailing/social media	8.7 (6.6, 10.8)***	0.26	56.4 (44.3, 68.6)***	0.77	1.1 (0.6, 1.6)***	0.16
Other internet use	4.6 (2.8, 6.4)***	0.15	32.2 (20.1, 44.2)***	0.44	0.2 (−0.3, 0.7)	0.03
Other PA^a^	15.4 (9.6, 21.2)***	0.16	12.0 (−1.6, 25.6)	0.15	16.0 (9.6, 22.4)***	0.16
Reading for leisure	−3.2 (−6.5, 0.0)	− 0.06	−1.0 (− 10.1, 8.1)	− 0.02	−3.6 (−7.1, − 0.1)*	− 0.07
Watching TV/movies	2.9 (−4.7, 10.6)	0.02	− 18.0 (− 38.0, 2.0)	− 0.15	6.3 (−2.0, 14.5)	0.05
Shopping	0.2 (−2.3, 2.6)	0.00	2.9 (−5.2, 11.0)	0.06	−0.3 (− 2.8, 2.3)	− 0.01
Music for leisure	0.8 (− 1.5, 3.1)	0.02	−3.5 (− 12.5, 5.5)	− 0.06	1.5 (− 0.8, 3.7)	0.04
Electronic gaming	2.5 (− 3.0, 8.0)	0.03	− 24.4 (− 43.3, − 5.4)*	− 0.21	6.7 (1.2, 12.3)*	0.08
Homework/study	8.8 (5.0, 12.5)***	0.14	4.3 (− 5.9, 14.4)	0.07	9.5 (5.5, 13.4)***	0.16
School lessons	9.0 (4.6, 13.4)***	0.12	9.2 (−0.5, 18.9)	0.16	9.0 (4.1, 13.8)***	0.12
Sleeping/napping	−5.6 (−11.7, 0.4)	−0.06	−15.5 (−33.5, 2.5)	− 0.14	−4.0 (− 10.4, 2.4)	−0.04
Daily living activities	11.9 (5.6, 18.2)***	0.12	16.9 (0.3, 33.6)*	0.17	11.1 (4.3, 17.9)**	0.11
Verbal communication	15.4 (11.7, 19.0)***	0.25	17.6 (8.0, 27.2)***	0.30	15.0 (11.1, 19.0)***	0.25

*n* number of participants, *CI* confidence interval

Bold text indicates statistically significant differences: *p < 0.05 **p < 0.01 ***p < 0.001 (paired-samples t-tests)

a.Among the analytic sample, other PA increased from 46.6 min/day at 11 years (95% CI = 43.1, 50.2) to 62.1 min/day at 13 years (95% CI = 57.3, 66.9). This increase was spread fairly uniformly across organized PA (+ 5.2 min/day, 95% CI = 0.8, 9.7), active transport (+ 5.0 min/day, 95% CI = 2.5, 7.6) and active chores/work (+ 5.2 min/day, 95% CI = 2.3, 8.1).

The characteristics of the segments are outlined in Table 5. In terms of sociodemographic characteristics, the two segments did not differ significantly in terms of sex, Indigenous status, language spoken at home or socioeconomic position. However, the ‘Social Screens’ were more likely to attend public school at age 13 (63.1%), compared with ‘the Mainstream’ (48.3%) (p < 0.01). On average, the ‘Social Screens’ also had higher scores on the pubertal development scale (2.5/4), compared with ‘the Mainstream’ (2.2/4) (*p* < 0.001), although subsequent analysis revealed that this was only the case among girls (p < 0.001). ‘Social Screens’ were also more likely than ‘the Mainstream’ to live in urban areas (90.9% versus 84.4%, *p* < 0.05). In terms of behavioural characteristics at age 13, ‘the Mainstream’ were more likely than the ‘Social Screens’ to participate in out-of-school activities in the last week (82.2% vs 74.6%, p < 0.05) and had higher mean PA participation (82.8 min vs 67.2 min, p < 0.05). No significant differences were observed in psychographic characteristics such as internalising, externalising, temperament and bullying victimisation. Finally, it should be noted that ‘the Mainstream’ were more likely to have been interviewed in winter in Wave 4 (54.2%) and the ‘Social Screens’ were more likely to have been interviewed in autumn in Wave 4 (37.1%) (both *p* < 0.01).

**Table 5:** Sociodemographic, geographic, psychographic and behavioral characteristics of clusters, LSAC data

Characteristics ^ab^	Cluster 1 (*n* = 143) (‘Social Screens’)	Cluster 2 (*n* = 900) (‘The Mainstream’)	Sig.
Sociodemographic/physical characteristics			
Sex (male), *n* (%) [w1]	66 (46.2%)	472 (52.4%)	0.162
Indigenous, *n* (%) [w1]	2 (1.4%)	21 (2.3%)	0.758^c^
Speaks a language other than English at home, *n* (%)	8 (5.6%)	70 (7.9%)	0.352
Socioeconomic position z-score, mean (SD)	0.0 (1.1)	0.1 (1.0)	0.485
Attends public school, *n* (%)	**89 (63.1%)**	**431 (48.3%)**	**0.001**
Number of siblings in household, mean (SD)	1.6 (1.0)	1.6 (1.0)	0.741
Child has two parents living at home, *n* (%)	119 (83.8%)	757 (84.1%)	0.926
Body mass index of child z-score, mean (SD)	0.4 (1.0)	0.3 (1.0)	0.577
Gross motor coordination scale, mean (SD)	1.8 (0.4)	1.8 (0.5)	0.942
Pubertal development scale, mean (SD)	**2.5 (0.8)**	**2.2 (0.8)**	**0.000**^**d**^
PEDS physical health scale, mean (SD)	82.8 (13.7)	84.6 (14.9)	0.179
Geographic characteristics			
Child lives in an urban area, *n* (%)	**130 (90.9%)**	**760 (84.4%)**	**0.042**
Psychographic characteristics			
SDQ internalising symptoms, mean (SD)	3.3 (2.9)	3.2 (3.0)	0.894
SDQ externalising symptoms, mean (SD)	3.9 (3.2)	3.9 (3.3)	0.799
SATI introversion scale, mean (SD)	2.6 (0.7)	2.6 (0.8)	0.783
SATI persistence scale, mean (SD)	3.6 (0.9)	3.6 (0.8)	0.827
SATI reactivity scale, mean (SD)	2.5 (0.9)	2.4 (0.8)	0.298
Child bullied at school in the last year, *n* (%)	40 (28.4%)	229 (26.2%)	0.588
Behavioral characteristics			
Child participated in any out-of-school activities in the last week, *n* (%)	**106 (74.6%)**	**739 (82.2%)**	**0.032**
Overall physical activity, mean min/day (SD)^e^	**67.2 (74.4)**	**82.8 (89.5)**	**0.047**
Season of measurement^f^			
Wave 4 - winter	**60 (42.0%)**	**488 (54.2%)**	**0.006**
Wave 4 - autumn	**53 (37.1%)**	**216 (24.0%)**	**0.001**

*n* number of participants, *%* proportion of sample, *w* wave of measurement, *SD* standard deviation

Chi-square tests (categorical variables) and independent t-tests (continuous variables), significant results are in boldface

a.Unless otherwise specified, the characteristics listed here were measured at Wave 5 (13y).

b.Variable-specific missing data: Sex (*n* = 0), Indigenous status (*n* = 0), language spoken at home (*n* = 10), socioeconomic position (*n* = 9), school type (*n* = 9), number of siblings (*n* = 1), number of parents in home (*n* = 1), body mass index (*n* = 11), gross motor coordination (*n* = 5), pubertal development (*n* = 7), PEDS physical health scale (*n* = 12), urban/rural status (*n* = 0), SDQ internalising (*n* = 12), SDQ externalising (*n* = 12), SATI introversion (*n* = 12), SATI persistence (*n* = 12), SATI reactivity (*n* = 12), child bullied at school (*n* = 28), child participation in out-of-school activities (n = 2), overall PA (n = 0), season of measurement (*n* = 0)

c.Fisher’s exact test performed because at least one cell had an expected value of less than 5.

d.When this result was tested separately for boys and girls, the difference between clusters was only significant for girls (F = 13.6, *p* < 0.001).

e.Overall PA was calculated as the total duration of time spent in non-organized PA, organized PA, active transport or active chores/work.

f.All seasons for both waves were tested, only statistically significant differences reported here.

## Discussion

This study sought to identify and describe segments of youth whose participation in non-organized PA declined between 11 and 13 years, based on changes in other activities during this period. Two segments were identified (‘Social Screens’ and ‘the Mainstream’), and these segments were found to have substantial internal consistency. ‘Social Screens’ were characterised by large increases in texting, emailing and social media use, and a moderate increase in other internet use. By contrast, ‘the Mainstream’ had less extreme increases spread across a wider range of activities, including other PA, homework/study and electronic gaming. In both segments, significant increases in participation were observed in verbal communication, daily living activities and texting, emailing and social media use. ‘Social Screens’ were more likely to attend public school, live in urban areas and have more advanced pubertal development (girls only). ‘The Mainstream’ were more likely to participate in physical activity and out-of-school activities.

Adolescence is a time of substantial physical, social and emotional change [28], and some adolescents withdraw from PA due to changing priorities or preferences during this stage of life [29, 30]. In the present study, both segments increased in their texting, emailing and social media use, although the increase was much more pronounced in the ‘Social Screens’ segment. Members of the ‘Social Screens’ segment were also less likely to engage in PA and out-of-school activities in general. This suggests that the ‘Social Screens’ segment may be at higher risk of inactivity during adulthood, as there is some evidence that PA participation during adolescence may track into adulthood [31]. This segment also has a similar profile to the high-risk ‘alternative’ peer subculture described by Jordan and colleagues [32] as taking pride in being different from the ‘mainstream’ [32] and being sceptical of overt health promotion approaches [33].

Although the ‘Social Screens’ segment is relatively small, it may be viewed as a niche subgroup that might be difficult to reach with broadly-targeted health promotion approaches [34]. Broad population approaches may therefore be complemented by specialized strategies targeted at potentially higher risk subgroups [34, 35]. For example, future PA interventions may adopt social marketing principles in order to replace the benefits that youth may be seeking in texting, emailing and social media [10]. Mobile phone technologies such as PA apps may be effective in motivating youth to be active by utilizing social comparison and peer approval [36]. One example is ‘inKin’, a social fitness app which enables users to compete with friends based on their PA level [37]. However, a greater emphasis on cooperation rather than competition may be preferred by youth who are at risk of becoming inactive [38]. This approach may be improved by involving socially influential adolescents known as ‘brand ambassadors’ [39], to overcome potential scepticism among the target audience. In the present study, the participants in the ‘Social Screens’ segment were more likely to live in an urban area and attend a public school. Therefore, an urban public school setting in Australia may provide a suitable setting to trial such a strategy. Further research may focus on developing and trialling approaches such as these.

It is notable that both segments in the present study had significant increases in verbal communication that were of similar magnitude. According to a systematic review of qualitative evidence, active youth often derive social interaction from their PA participation [38], while inactive youth often engage in inactive socialisation instead of PA [40, 41]. Youth who remain active during adolescence are also more likely to be motivated by personal mastery, whereas youth who become inactive tend to be motivated by extrinsic factors [38]. Potential extrinsic motivators for PA include affiliation, shared experience and positive social evaluation [42]. Therefore, PA promotion strategies may seek to replace the benefits that youth are seeking in verbal communication [10], by supporting physical activities that require verbal cooperation [43]. For example, Berstein and colleagues [43] described a lower-skilled student who enjoyed playing an improvised variation of the game of ‘tag’ that involved group strategy development. Such activities may be supported by loosely-facilitated PA sessions, similar to Högman and Augustsson’s ‘organized spontaneous sport’ model [44]. In this approach, a supervised program may be developed that sets a ‘safe’ culture for youth to engage in freely-chosen, improvised games that suit their interests and motives [44]. This might enable youth to pursue forms of PA that provide extrinsic, affiliative benefits [42].

In the present study, time spent ‘watching TV and movies’ did not increase significantly between 11 and 13 years in either segment, despite these activities being frequently used to define segments in previous cross-sectional studies [12, 14, 45, 46]. In the present study, texting, emailing, social media and other internet use were more likely than TV/movies to compete with non-organized PA across waves. This is consistent with the finding that younger generations use the internet more frequently and watch television less frequently than older generations [47]. Younger generations also have more positive attitudes toward internet advertising than older generations [47]. This highlights the increasing importance of ‘new media’, both as a potential competing behavior of PA and as a potential communication channel. In particular, the ‘Social Screens’ segment had a pronounced increase in texting, emailing, social media and other internet use, which was accompanied by a decrease in other forms of media use, such as electronic gaming. Mobile phone and online activities may have developed an element of salience among this segment, which is characterized by increasing absorption in a particular activity at the expense of other behaviors [48]. This indicates that online platforms may be particularly important for reaching this segment of youth.

It is also worth noting that the pubertal development of girls in the ‘Social Screens’ segment was more advanced than that of girls in ‘the Mainstream’ segment. There is evidence that early maturation is associated with increased internet use [49], and it has been suggested that youth are particularly vulnerable to social media influence due to extensive neural development that occurs during puberty [50]. This suggests that social media strategies may be effective in reaching early maturing adolescent girls, although messages must be carefully designed to highlight safe PA spaces for girls who may be self-conscious and fearful of judgement [51].

This was the first known study to use adolescent time-use data to explore the longitudinal competitors of non-organized PA participation. The longitudinal approach used in the present study allowed segments to reflect changes in activity participation, as opposed to static measures. This study also utilized detailed time-use data, which allowed the identification of specific activities of importance. However, the present study also had some limitations. Although other 24-h TUDs have been shown to collect valid [52] and reliable data [53], there may have been a degree of recall bias due to the self-reported nature of the data. This was attenuated via interviewer prompts and by having participants complete the diary throughout the day before the interview. In addition, although it was possible to control for whether or not the TUD was completed on a school day, it was not possible to control for the season of measurement. In addition, although psychometric data have been provided for some variables in Additional File 1, the validity and reliability of other measures was not known. Another limitation of the study was that the analytic sample size fell slightly short of 100 cases per variable, as recommended by Dolnicar and colleagues [54], although the final model still demonstrated substantial internal consistency. The sample size was also not sufficient to enable the testing of separate cluster solutions for boys and girls, although the two segments produced by the final model did not differ significantly by sex. It should also be noted that the present study has analysed a national sample of Australian youth, and results may not necessarily generalize to other contexts. Finally, the data used in this study were collected in 2010 and 2012, at a time when mobile phones and social media use may have been less common among the age groups included in the study.

## Conclusions

This study sought to identify and describe segments of Australian youth whose participation in non-organized PA declined between 11 and 13 years, based on changes in other activities during this period. Two segments were found (‘Social Screens’ and ‘the Mainstream’; κ = 0.66). Segments were distinguished by differences in the uptake of texting, emailing, social media and other internet use. The ‘Social Screens’ segment had a much larger increase in these activities and lower participation in overall PA and out-of-school activities, compared with ‘the Mainstream’. The ‘Social Screens’ segment were more likely to attend public school, live in urban areas and have more advanced pubertal development (girls only). Future strategies may seek to promote non-organized PA in Australia by seeking to replace the benefits that this segment may be seeking in competing behaviors. For example, PA apps may motivate youth to engage in non-organized PA by harnessing the social affiliative benefits of texting, emailing and social media use. Future research may further develop and trial such strategies, particularly in urban, public school settings.

## Supplementary information

**Additional File 1.** All descriptive variables.pdf’ - provides information about each descriptive variable, including the number of items, response categories, source, and validity and reliability information where available.

**Additional File 2.** Definition of PA domains.pdf’ – provides more information about the differences between domains of PA included in the present study.

**Additional File 3.** Populated STROBE checklist.pdf’ – provides the populated STROBE checklist requested as part of the submission process.

## Data Availability

The datasets analysed during this study are available in the LSAC Dataverse: https://dataverse.ada.edu.au/dataverse/lsac [55].

## Abbreviations

ANOVA: Analysis of variance
LSAC: Longitudinal Study of Australian Children
PA: Physical activity
SPSS: Statistical Package for the Social Sciences
TUD: Time Use Diary
TV: Television
