# Supplementary material for: ‘Social screens’ and ‘the mainstream’: longitudinal competitors of non-organized physical activity in the transition from childhood to adolescence
Source: Int J Behav Nutr Phys Act. 2020 Jan 14;17:5. doi: 10.1186/s12966-019-0908-0 (PMC7050814; doi:10.1186/s12966-019-0908-0)
Supplement: Supplementary file 1 — Additional File 1. All descriptive variables.pdf’ - provides information about each descriptive variable, including the number of items, response categories, source, and validity and reliability information where available. [file 12966_2019_908_MOESM1_ESM.pdf]

Additional File 1

Manuscript title: 'Social Screens' and 'the Mainstream': longitudinal competitors of non-organized physical activity in the transition from childhood to adolescence

Table A1. Variables used to explore the characteristics of time-use segments

Potential predictor (respondent)	Description
**Demographic/physical characteristics**	
Sex (P1)	Determined at Wave 1 [Male/Female]
Indigenous status (P1)	Whether the child is of Aboriginal or Torres Strait Islander origin [Yes/No]
Linguistic diversity (P1)	Whether the child speaks a language other than English at home [Yes/no]
Socioeconomic position of family (P1)	Scale derived by LSAC based on annual household income, educational attainment of parents and occupational status of parents. Validated against experience of economic hardship, being a recipient of income support and other variables (56)
Type of school child attends (P1)	Type of school the child attends [1. (Government) / 2. (Catholic) / 3. (Independent), categories 2-3 merged]
Number of siblings at home (P1)	The number of the study child's siblings living in the household.
2 parents in the home (P1)	Whether the study child has two parents living in the household [Yes/No]
BMI z-score (DO)	Standardized variable provided by LSAC [Weight(kg)/Height(m)^2]
Gross Motor Coordination (P1)	3 item scale: parent's assessment of how well the child can run, jump and balance compared to their peers [1. (Better than other children) / 2. (About the same) / 3. (Not as well as other children)]

Pubertal development (P1)	5 item scale: parent’s assessment of the child’s development of body hair, growth spurt, skin changes, facial hair (males), voice changes (males), breast changes (females) and menarche (females) [1. (Has not yet started) to 4. (Seems complete); menarche: Yes/No]. α = 0.77, validated against physician’s assessment (57)
PEDS physical health scale (P1)	8 item scale: frequency in the last month that the child had problems with physical tasks [1. (Never) to 5. (Almost Always)]; α = 0.88, validated against days of school missed due to health and other variables (58)
**Geographic variables**	
Urban/rural status (DO)	Derived by LSAC based on home address, based on the ‘Section of State’ classification within the ASGS [1. (major urban population) / 2. (other urban population) / 3. (bounded locality) / 4. (rural balance), categories 1-2 and 3-4 merged] (59)
**Psychographic variables**	
Internalising symptoms (P1)	Sum of emotional problems scale and peer problems scale from the SDQ [1. (Not True) / 2. (Somewhat true) / 3. (Certainly True)]. α: emotional symptoms (0.67), peer problems (0.57) (60)
Externalising symptoms (P1)	Sum of hyperactivity scale and conduct problems scale from the SDQ [1. (Not True) / 2. (Somewhat true) / 3. (Certainly True)]. α: hyperactivity (0.77), conduct problems (0.63) (60)
Introversion (P1)	4 item scale measuring willingness to approach new people from the SATI [1. (Never) to 5. (Always)]. α = 0.88, test-retest reliability = 0.82 (61)
Persistence (P1)	4 item scale measuring completion of tasks from the SATI [1. (Never) to 5. (Always)]. α = 0.90, test-retest reliability = 0.81 (61)
Reactivity (P1)	4 item scale measuring negative affect from the SATI [1. (Never) to 5.

	(Always)]. α = 0.90, test-retest reliability = 0.89 (61)
Bullying (P1)	Whether the child has been bullied at school in the last year [Yes/No]
**Behavioral characteristics of child**	
Participation in extracurricular activities outside school (P1)	Whether the child regularly participated in out-of-school activities in the last week (including community, sports, academic, religious, creative or skills-based activities) [Yes/No]
Overall physical activity participation (TUD)	Duration of time spent in non-organized PA, organized PA, active transport or active chores/work (time-use diary)

Acronyms (A-Z): ASGS = Australian Statistical Geography Standard; BMI = Body Mass Index; DO = direct observation; P1 = Primary responding parent; SATI = School-Age Temperament Inventory; SDQ = Strengths and Difficulties Questionnaire; TUD = time-use diary

References (not cited in manuscript)

56. Blakemore T, Strazdins L, Gibbings J. Measuring family socioeconomic position. *Aust Soc Policy*. 2009;8:121-168.

57. Petersen AC, Crockett L, Richards M, Boxer A. A self-report measure of pubertal status: Reliability, validity, and initial norms. *J Youth Adol*. 1988;17(2):117-133. doi: https://doi.org/10.1007/BF01537962.

58. Varni JW, Seid M, Kurtin PS. PedsQL™ 4.0: Reliability and validity of the Pediatric Quality of Life Inventory™ Version 4.0 Generic Core Scales in healthy and patient populations. *Med Care*. 2001;39(8):800-812. doi: https://doi.org/10.1097/00005650-200108000-00006

59. Australian Bureau of Statistics. Australian Statistical Geography Standard (ASGS): Volume 4 - Significant Urban Areas, Urban Centres and Localities, Section of State, July 2016 (Cat. No. 1270.0.55.004). 2017. http://www.abs.gov.au/ausstats/abs@.nsf/mf/1270.0.55.004. Accessed 7 September, 2018.

60. Goodman R. Psychometric properties of the strengths and difficulties questionnaire. *J Am Acad Child Adolesc Psychiatr*. 2001;40(11):1337-1345. doi: https://doi.org/10.1097/00004583-200111000-00015

61. McClowry SG. The Development of the School-Age Temperament Inventory. Wayne State University Press; 1995:271.
