# Supplementary material for: ‘Social screens’ and ‘the mainstream’: longitudinal competitors of non-organized physical activity in the transition from childhood to adolescence
Source: Int J Behav Nutr Phys Act. 2020 Jan 14;17:5. doi: 10.1186/s12966-019-0908-0 (PMC7050814; doi:10.1186/s12966-019-0908-0)
Supplement: Supplementary file 2 — Additional File 2. Definition of PA domains.pdf’ – provides more information about the differences between domains of PA included in the present study. [file 12966_2019_908_MOESM2_ESM.pdf]

Additional File 2

Manuscript title: ‘Social Screens’ and ‘the Mainstream’: longitudinal competitors of non-organized physical activity in the transition from childhood to adolescence

---

This file provides definitions of non-organized PA, organized PA, active transport and active chores/work, as well as the time-use diary codes used to measure each domain of PA.

Table B.1 Definition of PA domains included in the present study

**PA domain**	**Definition**	**LSAC time-use diary codes used in the present study**
Non-organized PA	“Freely chosen, unstructured ... flexible and spontaneous, minimally constrained by adult demands, there is no end product, no time pressures, no fear of failure and the activity appears to occur for its own sake” (7, p.25).	Ball games, riding bike/scooter/skateboard for leisure, skipping, running, chasing. (the overall term ‘unstructured active play’ was adopted in Wave 5).
Organized PA	“Usually involving training and competition, having a coach, and being organized by adults” (62, p.23) and “typically requiring specialised equipment, a special play area and time” (63, p.56).	Organized team sports and training (e.g. football, basketball, netball, cricket), organized individual sports and training (e.g. swimming, dancing, tennis, martial arts, gymnastics) [a].

PA domain	Definition	LSAC time-use diary codes used in the present study
Active transport	"Any form of human powered transportation (e.g. locomotion on foot, or bicycle, skateboard, etc)" (7, p.22).	Travel by foot, bike, scooter, skateboard, etc.<br>*Note: this is distinct from non-organized PA because it occurs for the purpose of 'travel', not 'leisure'.*
Active chores or work	"Household chores, including vacuuming/mopping, digging/planting, lifting/carrying" and "job tasks, including walking, lifting/carrying, and other activities of similar exertion" (64, p.265).	Activity codes for active chores/work were selected in a process described previously (6). Available codes were included in this domain if they were considered likely to exceed 3.0 metabolic equivalents (METs).<br>Activity codes in Wave 4 included: making own bed, tidying own room, cleaning, tidying other rooms, gardening, putting out bin, walking pets<br>Activity codes in Wave 5 included: cleaning/tidying, gardening/lawn mowing, walking/playing with pets, car washing, umpiring/refereeing, pamphlet delivering.<br>*Note: this domain is distinct from non-organized PA because it usually occurs for a purpose such as cleaning, home maintenance, paid work or pet care, rather than occurring for its own sake.*

LSAC = Longitudinal Study of Australian Children

a. Examples not included in Wave 5 codes.

References (cited in main article):

6. Kemp BJ, Parrish A, Batterham M, Cliff DP. Participation in domains of physical activity among Australian youth during the transition to adolescence: a longitudinal study. J Phys Act Health. in press

7. Active Healthy Kids Australia. Physical Literacy: Do Our Kids Have All the Tools? The 2016 Active Healthy Kids Australia Report Card on Physical Activity for Children and Young People. Available at: http://www.activehealthykidsaustralia.com.au/siteassets/documents/ahka-2016-summary_form-report-card.pdf. Accessed 27 June 2017.

Additional references (not cited in main article):

62. Hardy LL, King L, Espinel P, et al. NSW Schools Physical Activity and Nutrition Survey (SPANS) 2010: Full Report. Available at: http://www.health.nsw.gov.au/heal/Pages/spans-2010-full-report.aspx. Accessed 28 September 2017.

63. Department of Health. 2007 Australian National Children's Nutrition and Physical Activity Survey - User Guide. Available at: https://www.health.gov.au/internet/main/publishing.nsf/Content/589EFDBF5E7B916FCA257BF000211E08/$File/user-guide-v2.pdf. Accessed 1 September 2017.

64. Brownson RC, Eyler AA, King AC, et al. Patterns and correlates of physical activity among US women 40 years and older. *Am. J. Public Health* 2000; 90(2):264.
